# Representations of Subsecond Duration-Based Timing by Complex Spike Synchrony in Cerebellar Purkinje Neurons

**DOI:** 10.1523/JNEUROSCI.0219-26.2026

**Published:** 2026-06-29

**Authors:** Sheridan A. Goldstein, Spencer T. Brown, Indira M. Raman

**Affiliations:** ^1^Department of Neurobiology, Northwestern University, Evanston, Illinois 60201; ^2^Northwestern University Interdepartmental Neuroscience Program, Northwestern University, Evanston, Illinois 60201

**Keywords:** climbing fiber, GCaMP8f, high-speed imaging, inferior olive

## Abstract

The cerebellum contributes to associative motor learning and sensorimotor coordination in part by tracking subsecond time intervals between behaviorally relevant events, raising the question of how duration, or absolute time, is encoded. Here, we investigated whether information about duration is present in Purkinje cell complex spikes during repetitive sensory stimuli. Crus I Purkinje cells expressing the fast calcium indicator GCaMP8f were imaged at high speed (250 fps), allowing detection of complex spike-associated calcium signals from hundreds of Purkinje cell dendrites simultaneously, with 4 ms temporal resolution, in awake head-fixed mice of both sexes. Air puffs were applied to the whisker pad in stimulus trains that varied in the mean and variance of interstimulus intervals (ISIs, 100–900 ms). In responsive cells, the mean probability of complex spike firing increased approximately fivefold ∼35 ms post-puff, primarily owing to well-timed spiking after the stimulus rather than an increase in spike rate. The maximal response probability, and in some cells also the response latency, varied linearly with ISI. The values of both variables were consistent for each ISI, regardless of the attributes of the stimulus train, suggesting that they carried information about absolute, rather than relative, durations between stimulus pairs. Because each puff evoked only one or zero complex spikes per Purkinje cell, the dependence of spike probability on ISI emerged as a trial-by-trial dependence of the degree of synchronous firing on ISI, suggesting that subsecond absolute timing of somatosensory signals may be represented by complex spike synchrony across populations of Purkinje cells.

## Significance Statement

The cerebellum regulates motor behaviors that require tracking time intervals briefer than 1 s. Here, we tested how the complex spikes of cerebellar Purkinje cells may encode the interval duration between somatosensory stimuli. We find that at least two attributes of complex spiking vary linearly with the interval between pairs of stimuli, namely, firing likelihood and firing latency. Because of their low spiking probability, however, individual Purkinje neurons cannot accurately report timing information about single stimulus pairs. Nevertheless, the number and latency of complex spikes fired synchronously across the Purkinje cell population changed consistently with the absolute interval between somatosensory stimuli. These data thus demonstrate one way in which neurons may represent time.

## Introduction

To coordinate complex movement and instruct motor learning, the cerebellum must track duration-based or “absolute” time, as distinct from beat-based or “relative” time, between sensory events (Grube et al., 2010). For instance, when humans judge absolute temporal intervals during trains of auditory stimuli, FMRI reveals cerebellar activation (Teki et al., 2011), whereas patients with cerebellar damage show deficits in both internally and externally cued well-timed movements (Spencer et al., 2003; Thieme et al., 2013). Additionally, the cerebellum in humans, rabbits, and rodents is required for delay eyelid conditioning, which involves learned movements timed to occur at a fixed interval 100 ms–1 s after sensory cues (McCormick and Thompson, 1984; Perrett et al., 1993; Krupa and Thompson, 1997; Garcia et al., 1999; Nicolson et al., 2002; Jirenhed et al., 2007; Jirenhed and Hesslow, 2011, 2016; Heiney et al., 2014). Nevertheless, questions remain regarding how cerebellar neurons represent subsecond timing.

Purkinje cells, which participate in well-timed conditioned responses (Jirenhed and Hesslow, 2016), fire both simple and complex spikes. Simple spikes dominate the real-time output, with basal rates in awake mice of ∼70 spikes/s, reaching >100 spikes/s during movements (Sarnaik and Raman, 2018). Complex spikes, evoked by climbing fiber inputs from the inferior olive (IO), are ∼100-fold rarer, occurring at 1–2 spikes/s. Most rodent Purkinje cells are innervated by a single climbing fiber (Busch and Hansel, 2023), whose extensive synaptic contacts ensure that each IO action potential reliably elicits a complex spike (Llinás and Mühlethaler, 1988; Dittman and Regehr, 1998; Silver et al., 1998; Xu-Friedman et al., 2001). Complex spikes provide instructive signals for plasticity evoked by sensory stimuli and also occur spontaneously, owing to intrinsic properties of IO neurons, whose autonomous 1–10 Hz oscillations may contribute to time monitoring on subsecond scales (Llinás and Yarom, 1986; Jacobson et al., 2008; Llinás, 2011; Lefler et al., 2013). Despite the regular oscillations of IO cells in vitro, however, spontaneous complex spikes in vivo occur irregularly (Keating and Thach, 1995; Zempolich et al., 2021), raising the question of whether IO cell activity indeed translates into temporal representations in Purkinje cells in vivo.

Here, we performed high-speed, widefield imaging of climbing fiber-mediated responses in Purkinje cell dendrites of awake, head-fixed mice, while applying irregular and regular trains of air puffs with subsecond interstimulus intervals (ISIs) to the whisker pad. Complex spike probability transiently increased after air puffs, yet firing rates during the stimulation period remained similar to baseline, indicating that sensory responses were primarily well-timed complex spikes rather than additional spikes, thereby increasing population synchrony on time scales of ∼20 ms. Complex spike probability across populations of Purkinje cells varied directly with the absolute duration between any pair of consecutive puffs and were largely independent of relative time, i.e., the rate of puffs preceding or following the pair. In a subpopulation of topographically clustered Purkinje cells, complex spike latency also decreased with ISI. Thus, information about subsecond timing of somatosensory input is present in the degree and latency of complex spike synchrony across Purkinje cell populations.

## Materials and Methods

### Experimental animals

Experiments were performed in accordance with the institutional guidelines and approved by Northwestern University Institutional Animal Care and Use Committee. Mice were housed with standard care in an accredited veterinary facility, with *ad libitum* access to food and water. Experiments were performed on five female and three male Pcp2-cre+ mice (Jackson Laboratory #010536 or #004146) for labeling of Purkinje cells. Mice were 2–6 months old.

### Experimental design and statistical analysis

#### Surgeries

Mice were anesthetized with isoflurane (1–2%), and buprenorphine SR-LAB (1 mg/kg, s.c., ZooPharm) and meloxicam (20 mg/kg, s.c., Dechra) were administered preoperatively for analgesia. Lidocaine (2%) was injected under the scalp, and a headplate for head-fixation was implanted and secured with dental cement (C&B Metabond). To minimize motion artifacts during imaging, surgical microbind screws (1/16 SL; Fisher Scientific) were used to secure the headplate to the skull. A craniotomy was made over the center of lobule crus I, and 3–4 injections of 300 nl of virus encoding the fast calcium indicator jGCaMP8f (AAV9-CAG-flex-jGCaMP8f-wPRE, Addgene diluted 1:30, stock titer: 2.2 × 10^13^ GC/ml) were made at a depth of 600–625 μm. A coverslip (3 mm diameter, 0.15 mm thick; Harvard Apparatus) placed over the craniotomy was secured with dental cement and covered with Kwik-Cast (World Precision Instruments). After ∼2 weeks for recovery, mice were brought to the experimental setup to acclimate to head-fixation while sitting on a platform for 1–2 h each day for 1–3 d. Viral expression was adequate for imaging ∼4 weeks post-injection. Experiments were initiated after the acclimation period was complete and expression was sufficient.

#### Tactile stimulation

Air puffs (10 ms, 20 PSI) were controlled by a solenoid and applied through a 2 mm spout placed 1–2 cm from the center of the whisker pad ipsilateral to the cranial window. Patterned stimulus trains were generated by triggering the solenoid with voltage pulses from an Arduino. Stimulus triggers were recorded in pCLAMP (Molecular Devices), which permitted temporal alignment to the imaging frames.

“Regular protocols” consisted of 150 puffs, with each puff designated as a single “trial.” Puffs were applied with fixed ISIs of either 300, 500, or 700 ms, such that each trial was associated with a specific ISI. “Irregular protocols” consisted of 500 puffs of five different ISIs (range, 100–900 ms, incremented by 100 or 200 ms, depending on the experiment), with 100 trials per ISI. The order of ISIs was randomized, as confirmed with a Wald–Wolfowitz runs test returning *p* > 0.05 on sequences of 100 consecutive trials from each train. Each recording session included two regular protocols with different ISIs and two irregular protocols with different mean ISIs and/or different standard deviations (SD) on the ISI. In each session, irregular protocols were run before regular protocols with the same mean ISI.

#### Widefield, high-speed calcium imaging

Images were acquired on a one-photon microscope built in-house (Brown et al., 2025; Holla et al., 2026) either with a Kinetix sCMOS camera (Teledyne Photometrics) with a 16× 0.8 NA water immersion objective (Nikon) or with a Veo 610 sCMOS (Vision Research) camera with a 10× 0.45 NA air objective (Nikon). In all recordings, the final magnification was 5×.

Imaging was performed 4–12 weeks after viral injection. A field of view (FOV) in cerebellar crus I was illuminated with a 470 nm LED (M470L5, Thorlabs), via a 1,024 × 768 digital micromirror device (DMD, V-7000, Vialux) and acquired at 250 frames per second (fps), at either 16 bit (Kinetix) or 12 bit (Veo 610) resolution. The mean dimensions of the FOVs were 1,520 × 1,150 µm.

#### Whisker imaging

In addition to calcium imaging, whisker imaging was performed in two mice. All whiskers except C1 or C1 and C2 were trimmed on the pad contralateral to the side of the puff. A blue light (470 nm) illuminated whiskers from below, and whiskers were imaged at 250 fps with a Genie Nano M640 NIR camera (Teledyne DALSA). Voltage pulses were generated with a Master-8 to trigger and synchronize whisker imaging and calcium imaging, which were recorded in pCLAMP. The C1 whiskers were tracked using the Ridge Detection plugin in Fiji. Whisker angle was extracted in MATLAB and reported as angle from the midline.

#### Motion correction, preprocessing, and detection of Purkinje cell dendrites

Images from each FOV were motion-corrected with Suite2p (Pachitariu et al., 2017). To remove background fluorescence, a filtered frame was first generated by averaging or taking the minimum projection of a Gaussian-filtered version (SD = 30 pixels) of the first ∼50,000–80,000 frames. This averaged low-pass filtered frame was then subtracted from each of the raw frames from each FOV. Suite2p was then run on these preprocessed frames to extract regions of interest (ROIs) likely to correspond to the dendritic arbors of individual Purkinje cells. ROIs were manually curated to ensure that the datasets included only those with spatial footprints and fluorescence transients consistent with the known characteristics of individual Purkinje cell dendrites.

#### Fluorescence signal extraction and spike detection

Given the nature of 1-P imaging, several steps were taken to minimize cross talk across ROIs. Fluorescent calcium signals from each ROI were extracted in MATLAB only from pixels that did not overlap with those of other ROIs, by computing the frame-by-frame dot product between the spatial footprint of each ROI with each imaging frame. The raw fluorescence signal was then smoothed with a 20 ms sliding bin. To detect the time of onset of each calcium transient with high temporal precision, the first derivative was computed, and noise transients were attenuated using the MATLAB smoothdata function (adaptive bin size mean = 5 frames). Peaks were detected, and those with a peak prominence with a *z*-score > 2.3 were labeled as transients. To prevent misclassification of noise as transients, peaks with a duration of only 1 frame were excluded. The first frame in which the smoothed (two-frame) signal derivative of the calcium transient was positive was taken as the time of occurrence of the complex spike.

#### ROI inclusion/exclusion criteria

To maximize the likelihood that individual ROIs included in the analysis represented single Purkinje cell dendritic arbors, ROIs were included in the dataset only if they met the following criteria for all protocols tested: width <20 µm and length >30 µm, to be consistent with the dimensions of a single dendritic arbor; a firing rate of 0.5–4 Hz, to be within the range of single-cell complex spike rates; <1% of interspike intervals below 16 ms, to eliminate ROIs that might have contained spikes from multiple cells; and a signal-to-noise ratio >2 (mean ± SD, 6.2 ± 1.6). Across all ROIs in each FOV, the correlation between signals was 0.08 ± 0.06 (mean ± SD, measured in 12 ms bins from the regular protocol with 500 ms ISIs). In addition, analyses were done only on “responsive” Purkinje cells, defined as ROIs in which the response probability trace averaged across all stimuli had a *z*-score >1.96 (*p* *<* 0.05) occurring 20–96 ms after the puff, and a maximum response probability of at least 0.125 spikes/trial/24 ms rolling bin. Finally, to prevent potential ROI overlap between FOVs, only one FOV from each mouse was analyzed. In total, eight FOVs from eight mice met these criteria.

#### Analysis of complex spike-related fluorescence transients

Complex spike firing rates for each ROI were calculated as the total number of calcium transients detected during tactile stimulation, divided by the duration of the stimulus train. Baseline firing rates were calculated over the imaging period before (∼30 s) and after (∼30 s) each stimulus train.

Complex spike probability traces averaged across all trials (puffs) were generated for each ROI by binning spikes relative to stimulus time and dividing by the total number of trials, and the highest value 20–96 ms after the puff was taken as the maximum response probability. To estimate the appropriate window over which to compute the increase in spike probability, this analysis was repeated with bins ranging from 4 to 52 ms in increments of 4 ms, and maximum response probability was plotted against bin width. For small bin widths, probability increased approximately linearly with time window; as bin width increased, this relation became sublinear (Brown et al., 2025). For each bin width, the data were fitted with two straight lines, including points above or below each bin width value, and the sum squared errors for each fit were calculated. A bin width of 24 ms yielded the minimum error for the two fits and was selected for all further analyses. Bins were causal, such that the value at time 0 indicated events that occurred between −24 and 0 ms. Population complex spike probability traces were calculated by averaging the traces for all (or a specified subset of) ROIs in a FOV.

To analyze changes in complex spike firing over time during irregular protocols, complex spike probability traces for individual ROIs were averaged over 15 consecutive trials (regardless of ISI), which were defined as a “block.”

To calculate synchrony, event traces were first created for each ROI with a rolling 24 ms causal window stepped in 4 ms increments. The value in each window was set to 1 if a spike occurred and a 0 if not (Brown et al., 2025). Percent synchrony was then computed from all relevant ROIs in a FOV by summing the event traces and dividing by the total number of ROIs. Jitter analysis was conducted as in Herzfeld et al. (2023) and Brown et al. (2025). The time of each complex spike was jittered in time within the midpoints between it and the preceding and the following spike. Probability traces, percent synchrony, and peak synchrony were then calculated on the jittered data as above.

To quantify fluorescence signals, we estimated the baseline (*F*_0_) by examining the distribution of fluorescence in 1 s sliding bins (250 points) and taking as baseline for each timepoint the 8th percentile value from each 1 s bin (Dombeck et al., 2007). Inspection of the traces for percentiles from 5 to 15% verified that the 8th percentile values provided an adequate estimate of the baseline preceding each event. The *F*_0_ trace was subtracted from the raw fluorescence trace to obtain Δ*F*, which was then divided by *F*_0_ to obtain Δ*F*/*F*. For averaging, Δ*F*/*F* signals associated with each transient were aligned either to the onset of the puff or to the transient, as indicated.

Whisker position was calculated as the angle in degrees relative to the midline (tail, 0°; snout, 180°). Stimulus-evoked whisker movements were calculated by aligning the whisker position trace to puff times and averaging across trials. The change in whisker movement was calculated on the mean traces, by subtracting the minimum whisker angle from the maximum whisker angle after the puff. The SD of whisker deflections was calculated (100 ms bins). Trials with a SD >5° during the 52 ms window preceding the puff were defined as having pre-puff movement.

#### Statistical analysis

Data are reported as mean ± SEM except where noted as mean ± SD. Statistical significance was assessed with either paired or unpaired Student's *t* tests or two-sample Kolmogorov–Smirnov tests, as indicated, and *p* values are reported.

## Results

### High-speed, widefield calcium imaging of complex spikes in Purkinje cell dendrites

The importance of cerebellar signaling for well-timed, learned motor responses suggests that duration must be encoded within cerebellar circuits. Here, we sought to address the fundamental question of whether information about absolute time may emerge from inferior olivary input, even in the absence of explicit learning tasks. We therefore investigated whether timing information may be present, i.e., “represented,” within cerebellar spiking patterns, independently of whether such spikes might constitute a signal that is decoded for specific behaviors.

**Figure 1.**
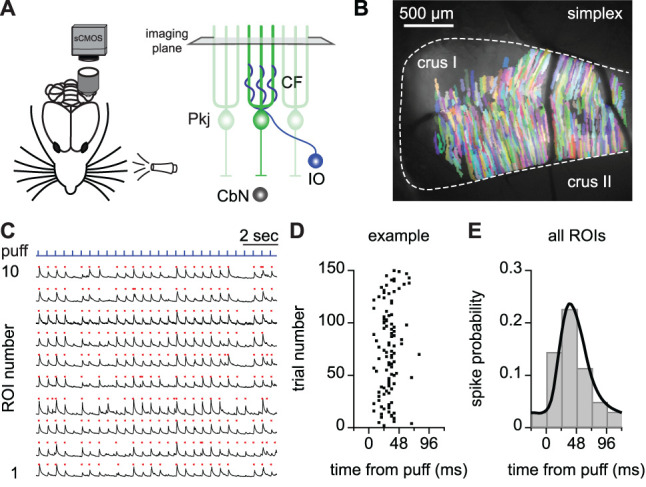
High-speed, widefield imaging of Purkinje cell dendrites. ***A***, Left, Schematic of experimental setup. Widefield calcium imaging of PcP2-cre Purkinje cell dendrites expressing AAV-FLEX-GCaMP8f was performed at 250 fps, while air puffs were delivered to the ipsilateral whisker pad of awake head-fixed mice. Right, Simplified diagram of key elements of the olivocerebellar circuit, including Purkinje cells (Pkj), an inferior olivary neuron (IO) with a climbing fiber (CF), and a neuron in the cerebellar nuclei (CbN). Gray shading denotes the imaging plane. ***B***, Sample FOV of crus I (dashed outline) with Purkinje cell dendritic ROIs individually colored (*n* = 529). ***C***, Blue, Segment of a train of puffs applied with 500 ms ISIs. Black, Sample normalized fluorescence traces from 10 ROIs from the FOV in ***B***. Red asterisks, Transients identified as complex spikes. ***D***, Raster of complex spikes from a single ROI in response to a train of 150 stimuli, applied as in ***C***. ***E***, PSTH (gray) with 24 ms bins for complex spikes averaged across all 3,187 ROIs in all 8 FOVs to the same protocol as in ***D***. Smoothed complex spike firing probability trace (black) is overlaid for the same data to illustrate the probability traces shown in all other figures.

Finding such representations requires temporally precise measurements of complex spiking across populations of Purkinje cells. We therefore conducted high-speed, widefield calcium imaging experiments, recording from dendrites of crus I Purkinje cells virally expressing the fast, genetically encoded calcium indicator, GCaMP8f (Zhang et al., 2023), in awake, head-fixed Pcp2-cre+ mice (*n* = 8 mice; Fig. 1*A*). Large groups of ROIs (range: 305–663 per FOV, total 3,990) were imaged at 250 fps (Fig. 1*B*). ROIs were 134 ± 61 µm long and 10 ± 3 µm wide (mean ±SD, *n* = 3,990), consistent with the expected morphology of Purkinje cell dendrites. Calcium transients were observed to occur both spontaneously and during regular trains of somatosensory stimuli applied to the whisker pad (10 ms air puffs applied with 500 ms ISIs; Fig. 1*C*). In Purkinje cell dendrites, such calcium transients have been shown to reflect responses to climbing fiber input (Ozden et al., 2009; Kitamura and Häusser, 2011; Roome and Kuhn, 2018). Consistent with this idea, in Purkinje cells that had a significant complex spike response to the puff, the mean basal rate in the absence of stimulation was 1.7 ± 0.5 transients/s (mean ± SD, *n* = 3,187 ROIs, 80% of total), the same as the basal complex spike rate of 1.7 spikes/s recorded electrophysiologically under comparable conditions (Zempolich et al., 2021; see also Chen et al., 2016). The imaged calcium transients thus serve as a good proxy for complex spikes and for simplicity are referred to as such, and ROIs are referred to as Purkinje cells.

The high imaging rate provided the advantage of 4 ms temporal resolution, which enabled precise detection of the onset of the rapidly rising GCaMP8f calcium transients (see Materials and Methods). To quantify complex spike timing relative to each puff, we first constructed peristimulus time histograms (PSTHs) with 24 ms bins for each ROI. Since Purkinje cells varied in the reliability with which they fired complex spikes after puffs (Fig. 1*D*), the complex spike probability across the population was obtained by averaging across all PSTHs (Fig. 1*E*, gray bars). To yield a continuous curve of population complex spike probability and facilitate illustration and quantification, the same data were plotted with a 24 ms sliding window in 4 ms steps (Fig. 1*E*, black line), referred to as the “probability trace.” The mean data showed an increase in complex spike probability that peaked 24–48 ms after the puff. This latency is consistent with electrophysiological studies (Zempolich et al., 2021), providing evidence that high-speed calcium imaging can accurately report the timing of complex spikes.

### Dependence of complex spike probability on absolute ISI

**Figure 2.**
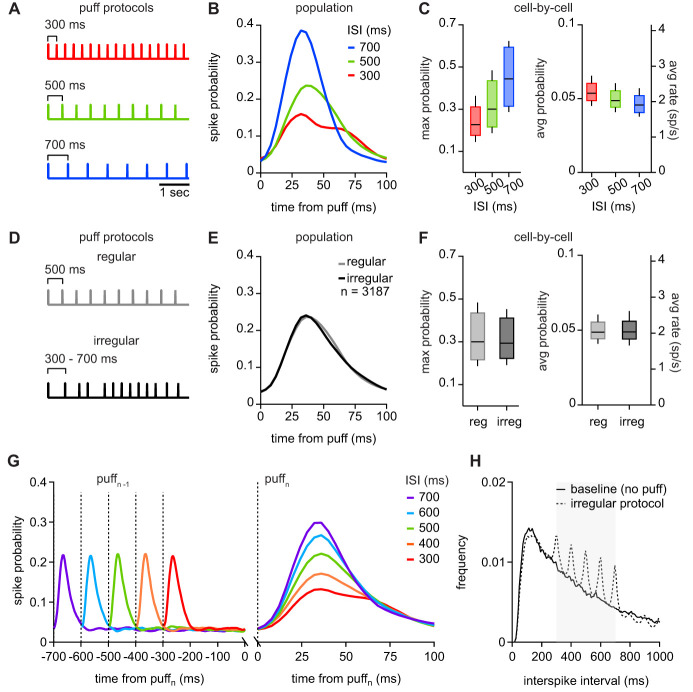
Complex spikes evoked by regular and irregular trains of somatosensory stimuli. ***A***, Schematic of the three regular protocols tested, with ISIs of 300 (red), 500 (green), or 700 (blue) ms. ***B***, Probability traces evoked by stimulus trains as in ***A***, averaged across the total population of cells imaged (ISIs of 300, 500, and 700 ms, *n* = 1,525; 3,187; and 1,662). ***C***, Left, Box-and-whisker plot of *P*_max_ from experiments in ***A*** measured on a per-cell basis to illustrate variance within the population. Black horizontal line, median; box, 25–75 interquartile range; whiskers, 1 SD. Right, Plot of complex firing rate averaged over the full stimulus period for each condition. ***D***, Schematic of a regular (gray) and irregular (black) protocol. Both have a mean ISI of 500 ms, but the latter is composed of 5 ISIs (300, 400, 500, 600, 700) applied pseudorandomly. ***E***, Probability traces evoked by stimulus trains as in ***D***, averaged across the total population of cells imaged (*n* = 3,187). ***F***, As in ***C***, for stimulus trains in ***D***. ***G***, Probability traces evoked by the irregular train in ***D*** with responses separated and averaged by ISI (*n* = 100 per interval), as labeled. The axis is split at the time of the puff (0 ms) to illustrate both the response to the preceding puff at left on a compressed time base (puff_n−1_), and the puff of the defined ISI of interest at right on an expanded time base (puff*_n_*). The amplitudes of puffs_n−1_ are comparable, because the ISI preceding them is a random mix of all intervals. ***H***, Frequency of interspike intervals (8 ms bins) of all complex spikes for all cells during a baseline period without puffs (solid line) and during the application of puffs in the irregular protocol (dotted line). Note that the most prevalent interspike intervals at baseline are between 100 and 200 ms and that the distribution of all interspike intervals is similar during stimulation, with the exception of increased probability of those matching the applied interstimulus intervals.

To test whether and how complex spiking varies with temporal attributes of sensory stimuli, we began by comparing responses to regular trains of 150 air puffs with ISIs of 300, 500, or 700 ms (Fig. 2*A*). As described above, puffs in trains with a 500 ms ISI elicited a peak spike probability (*P*_max_) of 0.24 ± 0.003 (*n* = 3,187; Fig. 2*B*). With the longer ISI of 700 ms, *P*_max_ increased to 0.39 ± 0.004 (*n* = 1,662). With the shorter ISI of 300 ms, *P*_max_ dropped to 0.16 ± 0.003 (*n* = 1,525), but the full probability trace broadened to include a later shoulder, suggestive of variable spike timing across the population. The data were therefore also analyzed on a cell-by-cell basis, in which the *P*_max_ evoked by the puff was measured from the mean probability trace for each Purkinje cell, regardless of when it occurred in the response window (20–96 ms after the puff). *P*_max_ increased steadily with ISI, from ∼0.25 to 0.45 (Fig. 2*C*, left, further quantified below). The firing probabilities measured over the full stimulation period for the ISIs of 300, 500, and 700 ms were 0.06 ± 0.01, 0.05 ± 0.01, and 0.05 ± 0.01 spikes/bin (mean ± SD; Fig. 2*C*, right). Converting these to rates gives 2.3 ± 0.4, 2.1 ± 0.4, and 2.0 ± 0.4 spikes/s during stimulation, all of which are only slightly higher than the basal firing rate of 1.7 spikes/s measured in the absence of puffs, before and after the train. This small increase in number of spikes generated cannot account for the four- to ninefold increase of puff-evoked *P*_max_ values relative to baseline. Therefore, the high *P*_max_ values must result from changes in spike timing rather than spike rate, with longer intervals leading to increased temporal precision with respect to the puff.

Owing to the constant ISIs, the puffs occurring during regular trains of stimuli are predictable, yet complex spiking is often associated with events that are unexpected, raising the question of whether stimulus regularity influenced *P*_max_. To test this possibility, we compared responses of the same Purkinje cell populations to regular trains of puffs with ISIs fixed at 500 ms (150 stimuli) and irregular trains of puffs with intervals of 300, 400, 500, 600, and 700 ms, with the same mean ISI of 500 ms (Fig. 2*D*). The irregular trains had 100 stimuli per ISI and were therefore longer than the regular trains. Controlling for train duration by analyzing only the first 150 stimuli indicated that puffs in both regular and irregular trains evoked similar patterns of complex spike firing, when responses were averaged across the full population (Fig. 2*E*). In cell-by-cell analyses, *P*_max_ values for regular and irregular trains were 0.34 ± 0.003 and 0.32 ± 0.002, (*n* = 3,187, *p* *<* *0.01*, paired *t* test; Fig. 2*F*, left) and occurred with a latency of 43.1 ± 0.3 and 43.0 ± 0.3 ms after the puff, respectively (*p* = 0.41, paired *t* test). Mean complex spike firing rates averaged over the full 150-puff stimulation period were 2.1 ± 0.4 (regular) and 2.1 ± 0.5 (irregular; mean ± SD, *p < 0.01*, paired *t* test; Fig. 2*F*, right). Thus, stimulus regularity per se had negligible effects on complex spiking.

To investigate whether complex spike probability might be sensitive to absolute interval duration, we separated puffs in the full-length irregular train according to ISI. Mean complex spike probability traces were computed by averaging responses to the 100 puffs per train for each of the five ISIs. The illustrated traces include the response to the preceding stimulus (puff_n−1_; Fig. 2*G*, left half) as well as the response to the stimulus that defined the ISI of interest (puff_n_; Fig. 2*G*, right half, on expanded time base). Even though the ISIs were presented in a train of pseudorandomly occurring intervals, complex spike P_max_ increased from 0.13 to 0.30 as ISIs became longer, much like in the regular trains with fixed intervals. We considered the possibility that, with progressively briefer intervals, the refractory period of IO neurons reduces the likelihood of climbing fiber firing, leading to lower *P*_max_ values in Purkinje cells. A histogram of intervals between spontaneous complex spikes recorded at baseline (without puffs), however, illustrates that the most frequently occurring interspike intervals were between 100 and 200 ms (Fig. 2*H*). Superimposing the interspike intervals during the stimulus period shows that the distributions largely overlay each other, with the exception of the increased probability at the stimulus intervals, which reflect well-timed spiking. These observations make it seem unlikely that the dependence of *P*_max_ on ISI is a consequence of the Purkinje cell or IO cell being unable to fire or generate complex spikes repeatedly with intervals as short as 100 ms.

**Figure 3.**
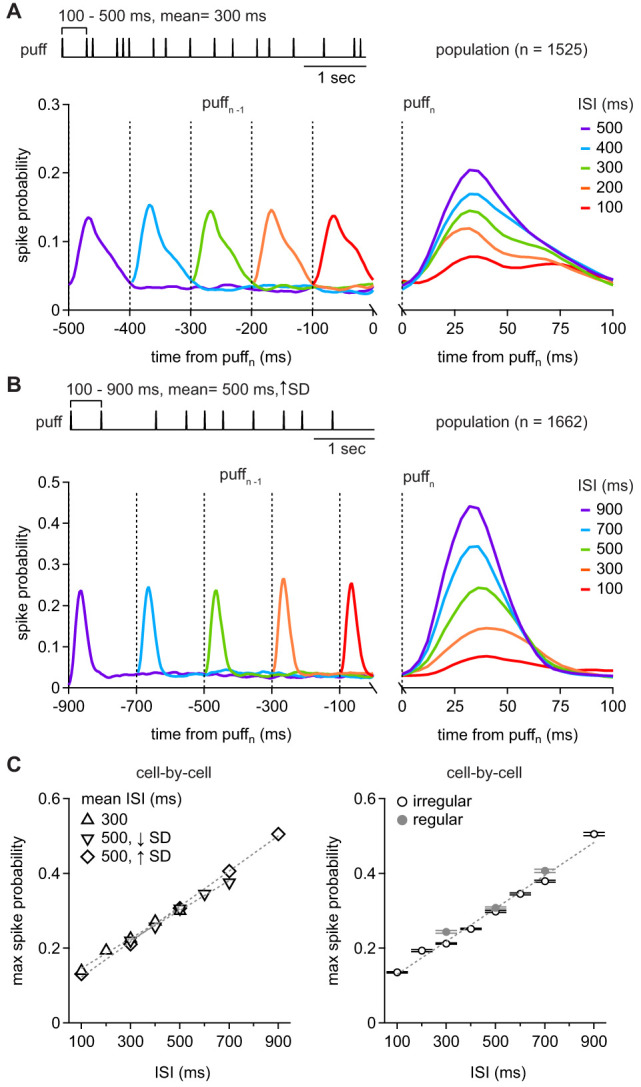
Dependence of complex spike firing probability on interstimulus interval. ***A***, Top, Schematic of irregular protocol with mean ISI of 300 ms and consisting of 5 different ISIs (100, 200, 300, 400, 500 ms). Bottom, Probability traces from the irregular stimulus train shown above, with responses separated and averaged by ISI (100 trials for each duration), as labeled (*n* = 1,525 cells). As in Figure 2*G*, the axis is split at the time of the puff (0 ms), with responses to puff*_n_*_−1_ on a compressed time base at left and to puff_n_ on an expanded time base at the right. ***B***, Top, Schematic of irregular protocol with mean ISI of 500 ms, consisting of five different ISIs (100, 300, 500, 700, 900 ms). Bottom, as in ***A***, for the stimulus train shown above (100 trials for each duration, *n* = 1,662 cells). ***C***, Left, Mean *P*_max_ for each irregular protocol versus ISI. Dashed lines, Linear fits to each dataset (*r* = 0.99 for all). Right, Mean *P*_max_ versus ISI, pooled for all irregular protocols (open, black circles) and regular protocols (closed, gray circles). Error bars, SEM. Dashed line, linear fit to the data from the irregular protocols (*r* = 0.99).

To evaluate whether and how complex spike probability varies with ISI across a broader range of intervals relevant to cerebellar timing, we tested two more stimulus protocols. The first was another irregular train of puffs with five intervals in 100 ms increments but extending to briefer ISIs (100, 200, 300, 400, 500 ms, with a mean of 300 ms). This pattern of stimulation demonstrated that *P*_max_ continued to fall with the shorter intervals of 200 and 100 ms (Fig. 3*A*). Additionally, the late shoulder on the mean probability trace for the smaller responses became more pronounced, indicative of a greater proportion of delayed complex spikes at the briefer intervals, considered further below. The second protocol was also irregular but had a greater standard deviation on the ISIs, which ranged from 100 to 900 ms in 200 ms increments (mean of 500 ms). With this train, *P*_max_ once more varied with ISI, reaching 0.44 with the longest interval (Fig. 3*B*).

To quantify the dependence of *P*_max_ on ISI, we repeated the cell-by-cell analyses to obtain the puff-evoked *P*_max_ for each cell. For each of the three irregular trains tested, this relationship was linear (*r* = 0.99 for each; Fig. 3*C*, left). Moreover, the *P*_max_ values for each ISI were consistent across protocols; for example, for an ISI of 500 ms, which existed in all stimulus trains, *P*_max_ fell between 0.30 and 0.31. The data were therefore averaged across protocols, which revealed a linear input–output relationship (*r* = 0.99) for peak complex spike probability versus ISI (Fig. 3*C*, right) extending across the range of known temporal intervals (100 ms–1 s) that can induce cerebellar learning. *P*_max_ nearly quadrupled from 0.14 ± 0.002 after the briefest ISI of 100 ms to 0.51 ± 0.004 after the longest ISI of 900 ms, giving a slope, *m*, of a 4.4% increase in maximal firing probability per 100 ms. Superimposing the *P*_max_ values from the ISIs in the three regular trains gave comparable values. These results demonstrate that *P*_max_ is largely independent of the temporal context of the stimulus, i.e., the mean, standard deviation, or regularity of ISIs within a train of events, supporting the idea that information about the absolute duration between sensory stimuli may be encoded in the probability of Purkinje cell complex spiking.

### Categorizing Purkinje cells by complex spike response latency

**Figure 4.**
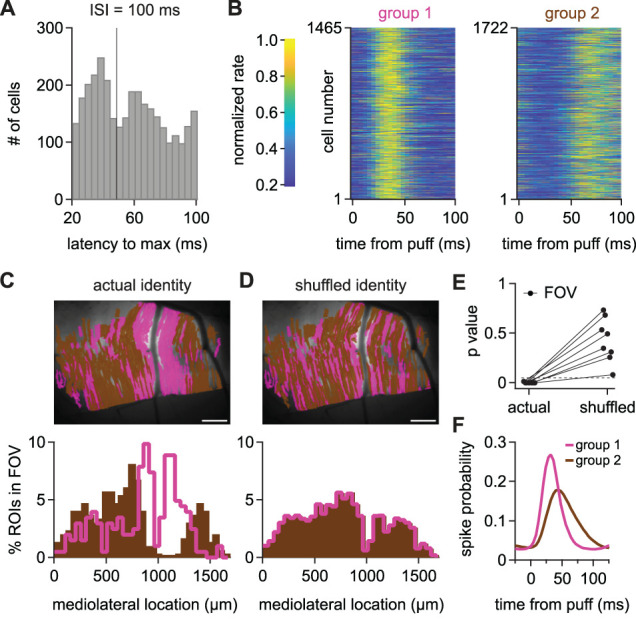
Grouping of Purkinje dendrites according to complex spike response latency. ***A***, Histogram of the latency to *P*_max_ for 100 ms ISIs in 3,187 cells. Vertical black line at 50 ms, separates ROIs into group 1 (latency to *P*_max_ <50 ms) and group 2 (latency to *P*_max_ > 50 ms). ***B***, Heat map of normalized complex spike probability traces of cells in group 1 (left, *n* = 1,465) and group 2 (right, *n* = 1,722). ***C***, Top, Sample FOV with group 1 ROIs in magenta (*n* = 203) and group 2 ROIs in brown (*n* = 295). Scale bar, 250 μm. Bottom, Histogram of the mediolateral locations of all ROIs in the sample FOV by group. ***D***, As in ***C***, but with group identity randomized by 1,000 shuffles. ***E***, Plot of *p* values from a two-sample Kolmogorov–Smirnov test comparing the distributions of group 1 and group 2 locations as in ***D*** for each of the 8 FOVs for the actual (*p* < 0.02 for all) and shuffled (*p* = 0.08 to 0.73) cases. Lines connect individual FOVs. Horizontal dashed line, *p* = 0.05. ***F***, Mean complex spike probability traces for group 1 and 2 cells in response to all puffs in all irregular protocols.

As noted above, the probability traces broadened at shorter intervals, indicative of increasing variability of spike times after briefer ISIs. To assess whether this temporal dispersion resulted from more jitter across the full population or from consistent responses from a subset of Purkinje cells, we examined the cell-by-cell latency of *P*_max_ after the shortest (100 ms) ISI, which was tested in all FOVs. A histogram of *P*_max_ latencies over the range from 20 to 96 ms showed two clear peaks, separated by a trough ∼50 ms (Fig. 4*A*). To investigate whether any qualitative differences in response patterns might exist across this distribution, ROIs were split into two categories, those with relatively early (< 50 ms, “group 1,” *n* = 1,465) or late (> 50 ms, “group 2,” *n* = 1,722) responses. This separation offered the convenience of yielding two comparably sized groups, both of which were consistently present in all FOVs, with proportions ranging from 20 to 80% (*n* = 8 FOVs). Plotting the probability traces for the 100 ms ISI for each Purkinje cell as a heat map further suggested that the complex spikes of cells in group 1 tended to occur within a relatively narrow time window, whereas group 2 fired over a broader time window (Fig. 4*B*).

When the spatial distribution of group 1 and group 2 cells were examined by color-coding them on the images of crus I, ROIs from the same group tended to form parasagittal clusters (groups 1 vs 2, mean mediolateral locations, two-sample Kolmogorov–Smirnov test; *p* < 0.02 for all recorded nonshuffled FOVs, “actual”; Fig. 4*C*,*E*). This nonrandom patterning was abolished when group identity was shuffled (*p* = 0.08 to *p* = 0.73 for “shuffled” FOVs, two-sample Kolmogorov–Smirnov test; Fig. 4*D*,*E*). Calculating probability traces separately for each group, but averaging over all ISIs tested, indicated that cells in group 1 had a shorter mean latency to *P*_max_ than group 2 (33.8 ± 0.24 vs 50.8 ± 0.39 ms, *p* < 0.001, unpaired *t* test; Fig. 4*F*). The two groups of Purkinje cells nevertheless had indistinguishable complex spike baseline firing rates during irregular protocols (group 1 vs group 2: 2.0 ± 0.41 vs 2.1 ± 0.42 spikes/s, mean ± SD, *p* *=* *0.7*, unpaired *t* test). Thus, although the initial categorization was based on a relatively coarse separation of latency histogram peaks for a single ISI, both the topography and the responses to all intervals support the idea that Purkinje cells produce at least two distinguishable patterns of complex spike responses.

**Figure 5.**
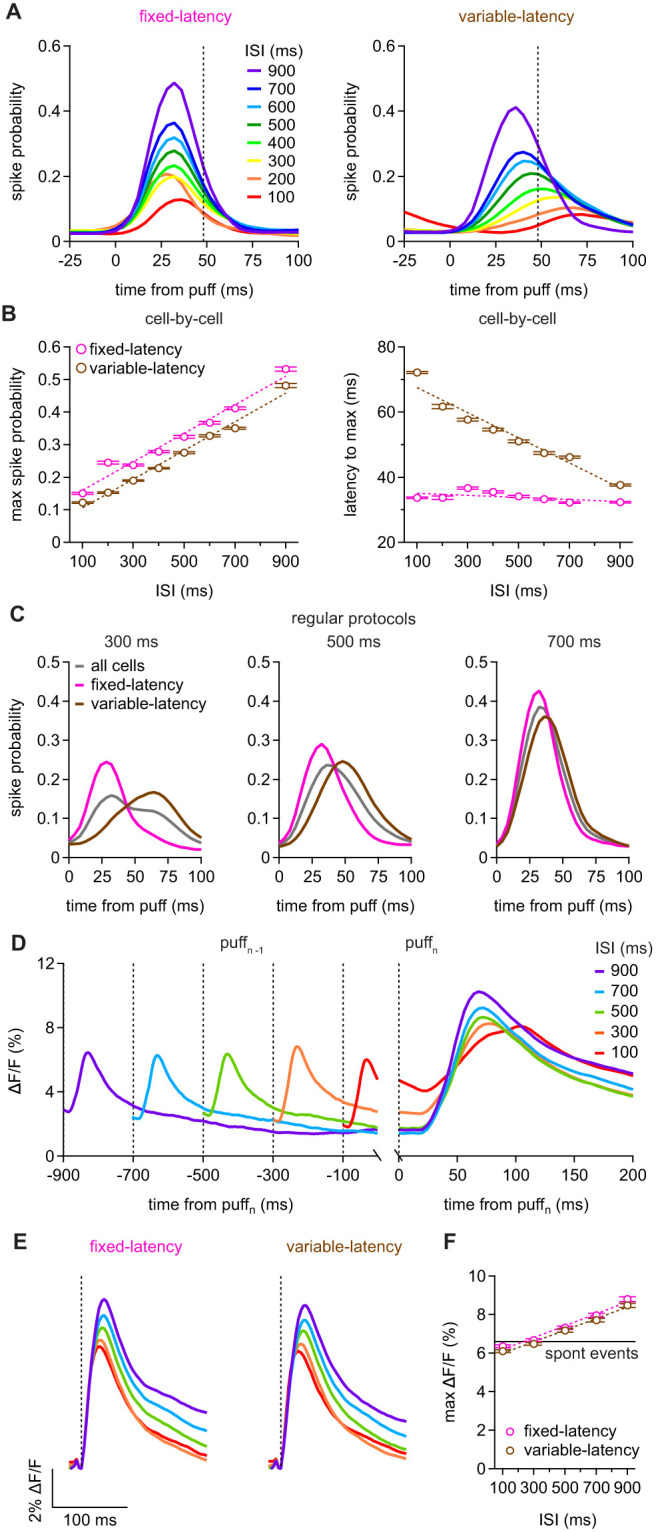
Distinct responses of fixed-latency and variable-latency Purkinje cells. ***A***, Left, Population probability traces of fixed-latency cells to puffs separated by ISI for all irregular protocols (*n* = 1,465 for ISIs of 100, 300, 400, 500, 600, and 700 ms, *n* = 670 for 200 ms ISI, *n* = 795 for 900 ms ISI). Right, Same as in ***A***, but for variable-latency cells (*n* = 1,722 for ISIs of 100, 300, 400, 500, 600, and 700 ms, *n* = 855 for 200 ms ISI, *n* = 867 for 900 ms ISI). ***B***, Left, Mean *P*_max_ of fixed- (magenta) and variable-latency (brown) cells from traces in ***A*** and ***B***. Dashed lines, linear fits (fixed- and variable-latency: *m* = 4.4% spike probability change per 100 ms increase in ISI, *r* = 0.99). Right, As in ***B***, left, for latency to *P*_max_ (fixed- vs variable-latency: *m* = −0.3 ms vs −3.9 ms per 100 ms, *r* = −0.56 vs −0.98). ***C***, Mean probability traces of all (gray), fixed- (magenta), and variable-latency (brown) cells to regular trains with ISIs of 300 ms (left, fixed-, variable-latency, *n* = 670, 855), 500 ms (middle, fixed-, variable-latency, *n* = 1,465, 1,722), and 700 ms (right, fixed-, variable-latency, *n* = 795, 867). ***D***, Mean Δ*F*/*F* from all cells tested with irregular stimulus trains with ISIs of 100, 300, 500, 700, and 900 ms, with responses separated and averaged by ISI, as labeled (*n* = 1,662 cells). The axis is split at the time of the puff (0 ms), with responses to puff*_n_*_−1_ on the left and to puff_n_ on the right. ***E***, Δ*F*/*F* zeroed and aligned to the time of transient onset, for fixed-latency cells (left, *n* = 795) and variable-latency cells (right, *n* = 867). ***F***, Max Δ*F*/*F* from ***E*** versus ISI for fixed- (pink) and variable-latency (brown) cells. Max Δ*F*/*F* in spontaneous transients in all cells (black). Dashed line, linear fit (*r* = 0.99 for both groups). Error bars, SEM.

We therefore reanalyzed the original datasets separately for the two groups, with additional attention to the latency of *P*_max_. In addition to the expected increase in *P*_max_ with interval, the probability traces of the population average for group 1 displayed a relatively constant latency to *P*_max_ (Fig. 5*A*, left). In contrast, the latency to *P*_max_ in group 2 steadily decreased for longer ISIs (Fig. 5*A*, right). We therefore renamed group 1 as “fixed-latency” and group 2 as “variable-latency” cells. Plotting *P*_max_ against ISI from the cell-by-cell analysis demonstrated similar linear relationships for fixed- and variable-latency cells (*m* = 4.4% spike probability increase per 100 ms increase in ISI; *r* = 0.99 for both), with the former having a slightly higher maximum firing probability across ISIs (probability difference between *y*-intercepts, 0.05; Fig. 5*B*, left). The dependence of *P*_max_ latency on ISI differed strongly, however (Fig. 5*B*, right). Fixed-latency cells showed a relatively flat relationship between latency to *P*_max_ and ISI (*m* = −0.3 ms per 100 ms increase in ISI, *r* = −0.56), with *P*_max_ consistently occurring between 32.3 and 36.7 ms, whereas in variable-latency cells, the relation was steep and negatively correlated (*m* = −3.9 ms per 100 ms increase in ISI, *r* = −0.98), with the latency to *P*_max_ falling from 72.2 to 37.7 ms. In response to regular trains of stimuli, the two groups also showed latency differences that were larger at shorter ISIs, which accounted for the temporal dispersion in the responses averaged over all ROIs (Fig. 5*C*). Thus, in a subpopulation of Purkinje cells, the latency to complex spiking also carries information about the time between stimuli.

Previous studies have demonstrated that calcium transients can themselves vary with stimulus parameters, including smaller responses for spontaneous complex spikes and larger responses for sensory-evoked complex spikes, which increase further with stimulus duration (Najafi et al., 2014a,b). Additionally, complex spike duration varies with the number of IO spikelets (Mathy et al., 2009). Therefore, to test whether the calcium signals themselves were affected by ISI, we examined the fluorescence traces directly. This analysis was restricted to the experiments in which the widest range of intervals was tested (100–900 ms, *n* = 1,662 ROIs in 3 FOVs) and demonstrated that peak calcium transients also increased with interval (Fig. 5*D*). For each ROI, responses were zeroed and aligned to the onset of the transient, rather than the time of the puff, and the peak ΔF/F was measured (Fig. 5*E*). The peak signal increased linearly with ISI (range, ∼6–9%), with responses that overlapped for fixed- and variable-latency cells (*m* = 0.3% Δ*F*/*F* per 100 ms, *r* = 0.99 for both; Fig. 5*F*). Across intervals, these values bracketed the peak Δ*F*/*F* elicited by spontaneous complex spikes in the absence of puffs (Δ*F*/*F* = 6.6%, with a mean spontaneous interspike interval = 595 ms; Fig. 5*F*). Thus, the size of the calcium signal within each cell may be yet another variable carrying information about stimulus interval.

### The effect of whisker movement on complex spike responses

**Figure 6.**
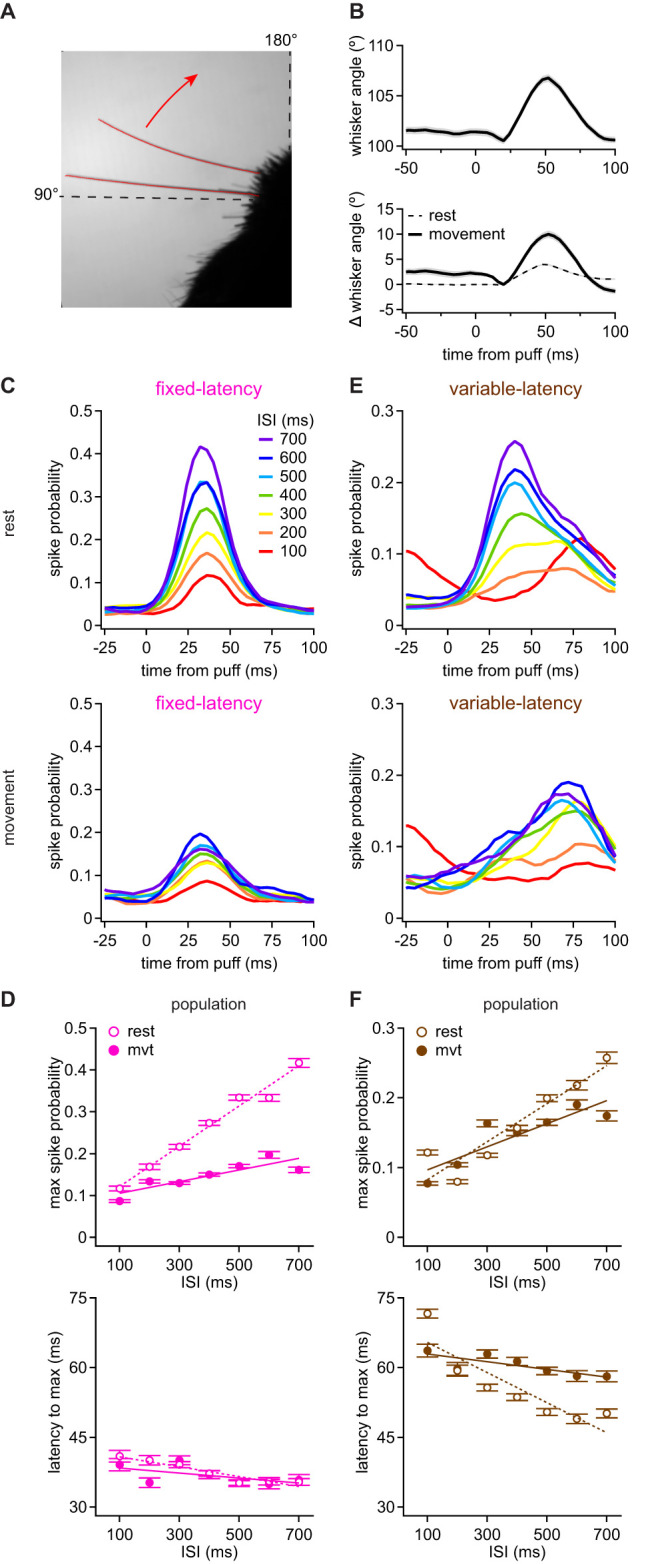
Effects of movement on complex spike modulation by ISI. ***A***, Dorsal view of mouse head, with whiskers C1 and C2 colored red. The C1 whisker was imaged at 250 fps and whisker angle was computed relative to the dashed lines indicating 90 and 180°. ***B***, Top, Mean whisker angle (black) ± SEM (gray) in response to all puffs in all irregular protocols (*n* = 2 mice, 2,000 trials). Bottom, Same data as above, separated for trials with whiskers resting (*n* = 1,090, dashed line) or moving (movement, *n* = 810, solid line) prestimulus. Gray shading, SEM. ***C***, Mean probability traces separated by ISI for fixed-latency cells (*n* = 174) for rest (top) and movement (bottom). ***D***, Top, Population *P*_max_ versus ISI for data from ***C*** for rest (open circles) and movement (closed circles). Lines, linear fits (rest vs movement: *m* = 4.8% vs 1.4% per 100 ms, *r* = 0.99 vs 0.86). Bottom, As above, for latency to *P*_max_ (rest vs movement: *m* = −1.1 vs −0.5 ms per 100 ms, *r* = −0.95 vs −0.57). ***E***, As in ***C***, for variable-latency cells (*n* = 316). ***F***, As in ***D***, for variable-latency cells. Top, Rest vs movement: m = 2.7% vs 1.7% per 100 ms, *r* = 0.93 vs 0.88). Bottom, Rest vs movement: *m* = −3.2 ms vs −0.8 ms per 100 ms, *r* = −0.89 vs −0.81.

In addition to being favored by sensory stimuli, complex spikes can be suppressed by ongoing movement (Ozden et al., 2012; Brown et al., 2024). In the present experiments, whisker protractions were reliably evoked by puffs, but mice also intermittently whisked spontaneously throughout the recording periods, raising the question of how such motor activity may have influenced complex spiking. In a subset of experiments, therefore, we performed high-speed imaging (250 fps) of the contralateral C1 whisker during widefield calcium imaging of Purkinje cell dendrites (Fig. 6*A*). When averaged across all stimuli in irregular trains, puffs elicited small, reflexive whisker protractions of 6.2° (Fig. 6*B*, top). When whisker position was analyzed for each stimulus trial, ongoing movement occurred ≤52 ms before the puff on 41% of trials (*n* = 810/2,000 puffs). On average, puff-evoked whisker protractions were larger when they were superimposed on spontaneous movement (10.0 ± 0.7°) than when they arose from rest (4.0 ± 0.1°, *p* < 0.001, unpaired *t* test; Fig. 6*B*, bottom).

We then separated complex spike probability traces based on whether the puff-evoked whisk was initiated when whiskers were resting or moving. Consistent with previous electrophysiological measurements (Brown et al., 2024), such volitional movement increased the complex spike firing rate preceding the puff (rest vs movement for 24 ms pre-puff: 1.5 ± 0.02 vs 2.3 ± 0.03 spikes/s, *n* = 490 cells, each in two conditions, *p* < 0.001, paired *t* test). For fixed-latency cells, response probabilities were far smaller on trials with the whisker initially moving than when it was resting, even though the sensory-evoked movements themselves were larger (Fig. 6*C*), and the steepness of the population *P*_max_ vs ISI relationship was reduced by movement (rest vs movement: *m* = 4.8% vs 1.4% per 100 ms, *r* = 0.99 vs 0.86; Fig. 6*D*, top). As expected, the latency to *P*_max_ was relatively stable with and without movement (Fig. 6*D*, bottom). In contrast, in variable-latency cells, movement greatly decreased the dependence of latency to *P*_max_ on ISI. Specifically, the difference between the extremes of the latency versus ISI relation fell from 21.4 ms (rest) to 5.7 ms (movement; Fig. 6*E*,*F*, bottom), while the slope of the *P*_max_ versus ISI relation was only moderately reduced (rest vs movement: *m* = 2.7% vs 1.7% per 100 ms, *r* = 0.93 vs 0.88; Fig. 6*F*, top). These data suggest that the ability of complex spikes to carry information encoding duration is highest in the absence of spontaneous movement.

### Trial-by-trial representations of ISI by complex spike synchrony

The above analyses reveal patterns relating absolute time and complex spike probability computed from multiple instances of the same interval within long trains of puffs, i.e., from averaging many trials. Complex spike probability cannot, of course, serve as a signal that can be decoded if trial-averaging is required. We therefore examined responses on a trial-by-trial (i.e., puff-by-puff) basis. Because complex spike responses to repeated stimuli are not always stationary (Ohmae and Medina, 2015; Bina et al., 2021; Zempolich et al., 2021), we began by examining the extent to which the puff-evoked *P*_max_ changed over the course of stimulus trains. *P*_max_ was averaged in blocks of 15 consecutive puffs, with data pooled for all irregular trains, regardless of the ISI.

**Figure 7.**
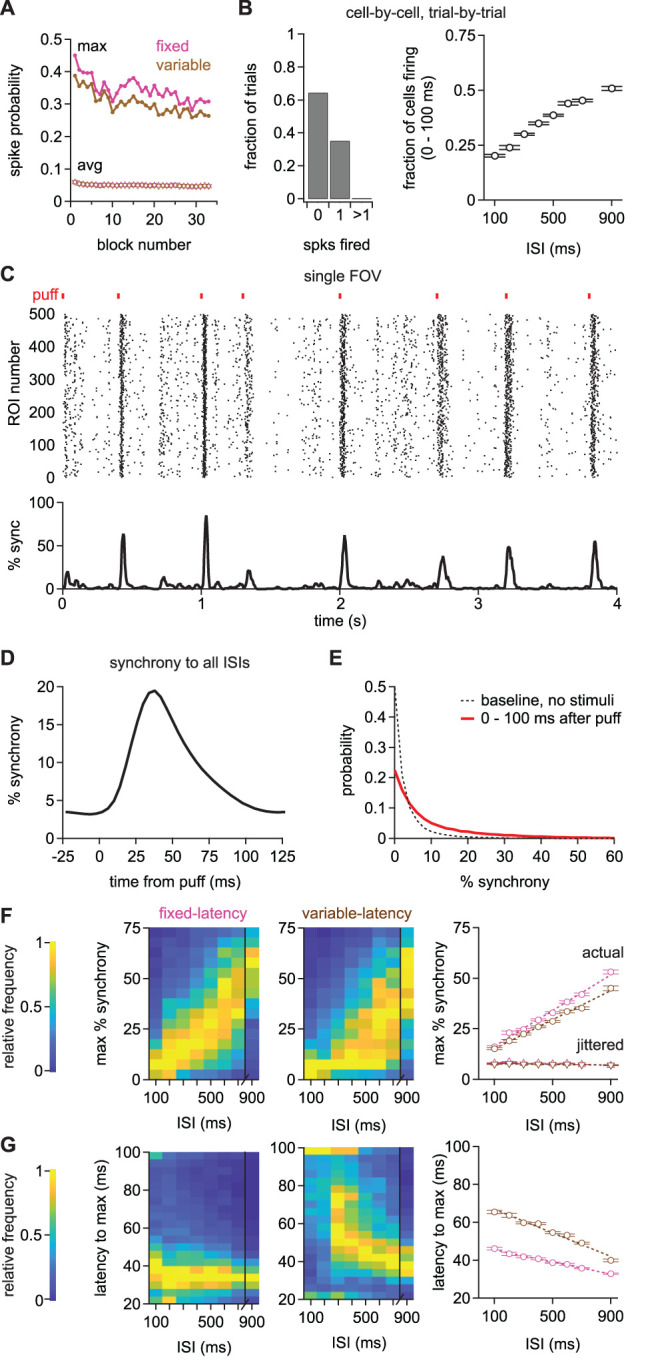
ISI-dependence of trial-by-trial complex spike synchrony. ***A**, P*_max_ (closed circles) and mean firing probability (open triangles) in 15-puff blocks, averaged across all irregular protocols, for all fixed- (magenta) and variable-latency (brown) cells. ***B***, Left, Fraction of trials in which cells fired 0, 1, or >1 complex spike in response to each puff for all irregular protocols. Right, Mean fraction of cells per FOV that responded to each ISI. ***C***, Top, Raster plot of complex spikes (black) from all cells imaged in a single FOV (*n* = 498) during an irregular train of puffs (red). Bottom, Percent of cells firing synchronously (24 ms rolling bin). Note stimulus-driven peaks in synchrony. ***D***, Mean complex spike synchrony in response to all puffs in all irregular protocols. ***E***, Probability distribution of complex spike synchrony (2% bins) occurring at baseline (dashed black line) and during the response period after the puff in all irregular trains (solid red line; *p* < 0.01, two-sample Kolmogorov–Smirnov test, 0–60% synchrony). ***F***, Heat map depicting the normalized frequency of occurrence of trial-by-trial peak percent synchrony for each ISI for fixed- (left) and variable-latency (middle) cells pooled across all irregular protocols. Vertical black line denotes break at 800 ms. Right, Mean peak synchrony for fixed- (magenta) and variable-latency (brown) cells, for actual (nonjittered, circles) and jittered (triangles) data. Dashed lines, linear fits (fixed-latency, actual vs jittered: 4.4% vs −0.16% per 100 ms increase in ISI, *r* = 0.99 vs −0.86; variable-latency, actual vs jittered: 3.6% vs −0.09% per 100 ms increase in ISI, *r* = 0.99 vs −0.88). Error bars, SEM. ***G***, As in ***F***, for the latency to peak synchrony (fixed- vs variable-latency: *m* = 1.6 ms, 3.1 ms per 100 ms increase in ISI, *r* = 0.99 for both).

In both fixed- and variable-latency cells, *P*_max_ fell over the duration of stimulation (first 5 vs last 5 blocks, fixed-latency: 0.41 ± 0.14 vs 0.30 ± 0.13, *n* = 2,930, variable-latency: 0.37 ± 0.11 vs 0.27 ± 0.10, *n* = 3,444, both *p* < 0.01, paired *t* tests), with the bulk of the decrease occurring within the first 150 stimuli (Fig. 7*A*). These time-dependent decrements likely account for the slightly higher *P*_max_ values measured above for the regular trains of stimuli, which had only 150 puffs per train. The average spike rate measured throughout the train in 15-puff blocks, however, remained stable at ∼2 spikes/s (Fig. 7*A*), again consistent with the idea that the stimulus preferentially sets the timing of ongoing spikes rather than eliciting additional spikes, a process that evidently degrades slightly with repeated stimulation.

Indeed, across all ISIs in the irregular trains, each Purkinje cell either fired a single complex spike time-locked to the puff or did not fire at all. Even in responsive cells, fully 64.4% of puffs evoked no complex spike, while 35.3% of puffs evoked a single complex spike, and only 0.3% of puffs evoked more than 1 spike (Fig. 7*B*, left). This nearly binary response prevents individual cells from encoding time on a trial-by-trial basis, because a single complex spike in a single cell cannot uniquely represent a particular ISI. Although the amount of calcium influx provides a potential single-cell signal, it is associated with the number of IO axonal spikes, which ranges from 1 to 5 (Mathy et al., 2009) and is unlikely to represent intervals with high resolution. Instead, the fraction of the population generating a complex spike on any given trial varied consistently with ISI, changing 2.5-fold from the briefest to the longest ISI (100 and 900 ms ISIs, 0.20± 0.006, *n* = 800 trials, and 0.51 ± 0.009, *n* = 300 trials; Fig. 7*B*, right), suggesting that a population code may be able to carry trial-by-trial information about absolute time.

Therefore, to examine population responses without averaging across puffs, complex spike rasters were generated for all ROIs in each FOV, which illustrated on a trial-by-trial basis the well-timed firing that contributed to the high *P*_max_ values described above (Fig. 7*C*, top). Summing the events per 24 ms bin and dividing by the number of cells generated a percent synchrony trace (Brown et al., 2025; Fig. 7*C*, bottom). Much like the probability traces (computed for each cell and averaged), the per-trial percent synchrony trace (computed for each trial and averaged) rose and fell from ∼3 to 20% (baseline to maximum; Fig. 7*D*). Complex spike synchrony was occasionally observed independently of the applied puffs; however, comparing all-points frequency histograms of percent synchrony values in baseline (nonstimulus) periods and the 100 ms window following the puffs demonstrated that stimulus-related synchrony significantly exceeded the synchrony in baseline periods (*p* < 0.01, two-sample Kolmogorov–Smirnov test, 0–60% synchrony, bin width 2%; Fig. 7*E*). Moreover, unlike the all-or-none spike responses of individual neurons, puff-related complex spike synchrony across the population was a graded variable.

We therefore analyzed the magnitude and timing of trial-by-trial complex spike synchrony across FOVs. In both fixed- and variable-latency cells, the percent synchrony increased with ISI, as is evident in the heat map of the relative frequency of the peak synchrony values per trial for each ISI (Fig. 7*F*, left, middle). The plot of mean peak synchrony versus ISI showed a strong and steep linear relationship in both cell groups (fixed-, variable-latency; *m* = 4.4%, 3.6% per 100 ms, *r* = 0.99, 0.99; Fig. 7*F*, right). To estimate the synchrony arising from any changes in firing rate, we conducted a jitter analysis. The peak synchrony calculated from the jittered spike times ranged from 7.0 to 8.7% across ISIs, all of which were lower than the synchrony of the raw data at all ISIs. Moreover, the jittered values did not increase with ISI (fixed-, variable-latency; *m* = −0.16%, −0.09% per 100 ms).

In fixed-latency cells, the time to maximal synchrony changed by 1.6 ms per 100 ms (*r* = −0.99), whereas in variable-latency cells, the slope was steeper, falling by 3.1 ms per 100 ms (*r* = −0.99; Fig. 7*G*). The magnitude and latency of trial-by-trial synchrony of complex spikes across a population of Purkinje cells are thus variables suited to encode absolute subsecond timing of sensory stimuli.

## Discussion

The present experiments demonstrate that repetitive air puffs applied to the whisker pads of awake mice elicit Purkinje cell complex spike responses with attributes that vary systematically with subsecond somatosensory stimulus intervals. Cell-by-cell analyses of calcium signals recorded in hundreds of Purkinje cells simultaneously, with high temporal resolution, reveal that the probability of well-timed complex spiking across trials increases linearly with the duration between consecutive puffs. Additionally, in about half the Purkinje cells, the response latency falls systematically with ISI. Moreover, for any given ISI, the puff-evoked complex spike probability is context independent, remaining relatively consistent regardless of the mean or standard deviation of the intervals of the train in which it is presented. Trial-by-trial analysis, however, demonstrates that each Purkinje cell fires only 1 or 0 complex spikes after each puff, indicating that stimulus interval is unlikely to be encoded by individual neurons on a single-trial basis. Instead, information about absolute subsecond interval emerges as a population code, in which the magnitude and/or latency of complex spike synchrony can vary directly with ISI.

### Complex spike firing evoked by stimulus trains

Puffs induce an increased probability of firing complex spikes in responsive Purkinje cells as early as ∼35 ms post-stimulus (Bosman et al., 2010; Brown and Raman, 2018; Brown et al., 2024). Repetitive stimulation with puff intervals of 100–900 ms demonstrates that this probability increase is greatest with long ISIs and falls linearly with decreasing intervals. This pattern is unlikely to arise solely from refractory periods reducing responses to more closely timed puffs, since interspike intervals of 100–200 ms predominate among spontaneous complex spikes; similarly brief interspike intervals can be evoked by direct stimulation in anesthetized rats (Marshall and Lang, 2004). Thus, the systematically varying *P*_max_ values seen here likely result from sensory stimulation eliciting specific firing patterns in IO neurons rather than only from intrinsic properties.

Previous studies indicate that complex spike latencies can vary, either within cells with different stimuli or across cells with the same stimulus (Bosman et al., 2010; Zempolich et al., 2021); even with constant (2 s) ISIs, complex spike probability and response latency are negatively correlated in anesthetized rats (Brown and Bower, 2001). The present data, however, identify a subset of variable-latency Purkinje cells in which briefer intervals yield systematically longer latency *P*_max_ values. Variable- and fixed-latency cells each composed about half the Purkinje neurons imaged, and both types were spatially organized in all FOVs. This organization is unlikely to be zebrin related, since crus I is predominately zebrin positive (Sugihara and Shinoda, 2004, 2007; Sugihara and Quy, 2007). Purkinje cells within each group are nevertheless located near each other in parasagittal bands, as predicted by the existence of microzones of Purkinje cells innervated by common regions in the IO (Sugihara and Shinoda, 2004; Ozden et al., 2009; Schultz et al., 2009; de Gruijl et al., 2014; Kostadinov et al., 2019; Tang et al., 2019; Michikawa et al., 2021).

Although complex spikes can influence real-time motor output (Welsh et al., 1995; Kitazawa et al., 1998; Tang et al., 2019; Tsutsumi et al., 2020; Gaffield et al., 2022), movement itself can affect complex spiking, often greatly reducing spike probability (Ozden et al., 2012; Brown et al., 2024), possibly by activating nucleo-olivary cells that inhibit IO neurons (Fredette and Mugnaini, 1991; Best and Regehr, 2009; Lefler et al., 2014). In the present study, in which mice were not engaging in learned motor tasks, even spontaneous whisking suppressed complex spiking. The consequent drop in *P*_max_ decreased the dependence of complex spike probability on ISI, raising the possibility that certain movements may reduce complex spike synchrony and, consequently, the accuracy of IO-dependent temporal representations.

### Complex spike synchrony and IO-driven coding of absolute time

As in anesthetized rats with low-frequency tactile stimulation (ISIs >1 s; Schultz et al., 2009), the post-puff increases in complex spike probability seen here are not strongly driven by the generation of more spikes. Even when puff rate more than doubles (e.g., 700 ms vs 300 ms ISIs), firing rates do not appreciably increase. Instead, stimulation primarily leads spikes to occur in a fixed temporal window (Zempolich et al., 2021), generating synchronous firing across the population.

Complex spike synchrony has been reported frequently (de Zeeuw et al., 2011), albeit with widely ranging time scales. For example, baseline complex spike synchrony with 1 ms precision is present in anesthetized rats (Sasaki et al., 1989), likely in Purkinje cells innervated by a common climbing fiber. Sensory input such as optokinetic stimulation can increase synchrony on a comparable timescale in pairs of Purkinje cells in anesthetized rabbits (Wylie et al., 1995). Calcium imaging studies that target larger groups of cells but with lower temporal resolution (100 s of ms) reveal sensory-induced complex spike synchrony in anesthetized and awake rodents (Schultz et al., 2009; de Gruijl et al., 2014; Najafi et al., 2014a,b). Finally, in awake rodents, electrophysiological recording from single Purkinje cells reveals well-timed complex spikes following sensory stimuli, indicative of population synchrony (Bosman et al., 2010; Ohmae and Medina, 2015; Heffley et al., 2018; Zempolich et al., 2021; Brown et al., 2024). The existence of widespread, stimulus-evoked complex spike synchrony suggests that synaptic input from sensory signals organizes activity in IO neurons, such that groups of climbing fibers fire together (de Zeeuw et al., 2011).

Much evidence supports the idea of such coordinated activity among IO cells, facilitated by gap junctions, as well as by intrinsic properties that favor synchronization (Yarom and Cohen, 2002; Lang, 2003; Lefler et al., 2013; Schweighofer et al., 2013). Specifically, membrane potentials of many IO neurons intrinsically oscillate at 1–10 Hz in vitro (Llinás and Yarom, 1981, 1986; Bal and McCormick, 1997; Lampl and Yarom, 1997), a period consistent with the time scale of cerebellar associative learning (Mauk et al., 1986; Jirenhed and Hesslow, 2016). Owing to the changing membrane potential, IO spike properties vary across the oscillatory cycle (Mathy et al., 2009; Bazzigaluppi et al., 2012; Sultan et al., 2025). Moreover, electrotonic coupling can bring oscillations into phase with one another and coordinate spiking across the network (Llinás et al., 1974; Llinás and Yarom, 1981; Long et al., 2002; Marshall et al., 2007). Such observations have led to the proposal that IO cells may constitute a neural clock that temporally coordinates cerebellar responses (Llinás and Yarom, 1986; Yarom and Cohen, 2002; Jacobson et al., 2008; Llinás, 2009, 2011; Lefler et al., 2013). Most studies relating IO oscillations to Purkinje complex spiking in vivo, however, have been done in anesthetized animals (Llinás and Sasaki, 1989; Brown and Bower, 2002; Chorev et al., 2007; Khosrovani et al., 2007), and whether the regular in vitro oscillations dictate in vivo spike timing is questioned based on the irregularity of complex spikes in awake animals (Keating and Thach, 1995). Moreover, each IO neuron only fires every 1–2 s, which theoretically limits the temporal resolution that can be relayed to the cerebellum, although higher precision may arise from coupled activity across the network (Yarom and Cohen, 2002; Ozden et al., 2009; Lefler et al., 2013). Regardless of the specific contribution of oscillations, electrical coupling of the IO network is crucial for olivocerebellar function, since loss of gap junctional proteins impedes cerebellar motor learning and disrupts complex spike synchrony (Marshall et al., 2007; van der Giessen et al., 2008; Ozden et al., 2009; de Gruijl et al., 2014).

The present results suggest a possible coding mechanism linking these observations by providing evidence that information about subsecond timescales is present in both the magnitude and latency of synchronous complex spikes across the Purkinje population. Whether and how such information is used remains a question. Given the instructional role of complex spikes in synaptic plasticity (Kistler et al., 2000; Ito et al., 2014; Hull, 2020), one possibility is that the efficacy of learning correlates with the number of complex spikes across the population, such that the lower synchrony found at briefer intervals between complex spike-eliciting stimuli would generate poorer learning. Indeed, in delay eyelid conditioning and VOR plasticity, learning is less effective with brief intervals or high-frequency stimuli (Mauk et al., 1986; Raymond and Lisberger, 1996). In eyeblink conditioning, however, the temporal precision of conditioned responses decreases again at long intervals (Heiney et al., 2014), indicating that learning is not a simple monotonic function of complex spike probability.

As complex spike synchrony increases, the informational content transmitted downstream decreases, as with any correlated signal (Schultz et al., 2009). The present data, however, suggest that, for stimulus pairs on time scales relevant for cerebellar learning, the degree of correlation may itself be information, which may report interstimulus timing. Thus, an alternative, not mutually exclusive, possibility arises from the idea that the degree of complex spike synchrony sets the extent of parallel fiber depression across the population. If the extent of plasticity were to dictate response latency, then the degree of complex spike synchrony could permit learning of the absolute time between associated stimuli.
